# A snapshot of antimicrobial resistance in Mexico. Results from 47 centers from 20 states during a six-month period

**DOI:** 10.1371/journal.pone.0209865

**Published:** 2019-03-26

**Authors:** Elvira Garza-González, Rayo Morfín-Otero, Soraya Mendoza-Olazarán, Paola Bocanegra-Ibarias, Samantha Flores-Treviño, Eduardo Rodríguez-Noriega, Alfredo Ponce-de-León, Domingo Sanchez-Francia, Rafael Franco-Cendejas, Sara Arroyo-Escalante, Consuelo Velázquez-Acosta, Fabián Rojas-Larios, Luis J. Quintanilla, Joyarit Y. Maldonado-Anicacio, Rafael Martínez-Miranda, Heidy L. Ostos-Cantú, Abraham Gomez-Choel, Juan L. Jaime-Sanchez, Laura K. Avilés-Benítez, José M. Feliciano-Guzmán, Cynthia D. Peña-López, Carlos A. Couoh-May, Aaron Molina-Jaimes, Elda G. Vázquez -Narvaez, Joaquín Rincón-Zuno, Raúl Rivera-Garay, Aurelio Galindo-Espinoza, Andrés Martínez-Ramirez, Javier P. Mora, Reyna E. Corte- Rojas, Ismelda López-Ovilla, Víctor A. Monroy-Colin, Juan M. Barajas-Magallón, Cecilia T. Morales-De-la-Peña, Efrén Aguirre-Burciaga, Mabel Coronado-Ramírez, Alina A. Rosales-García, María-de-J. Ayala-Tarín, Silvia Sida-Rodríguez, Bertha A. Pérez-Vega, América Navarro-Rodríguez, Gloria E. Juárez-Velázquez, Carlos Miguel Cetina-Umaña, Juan P. Mena-Ramírez, Jorge Canizales-Oviedo, Martha Irene Moreno-Méndez, Daniel Romero-Romero, Alejandra Arévalo-Mejía, Dulce Isabel Cobos-Canul, Gilberto Aguilar-Orozco, Jesús Silva-Sánchez, Adrián Camacho-Ortiz

**Affiliations:** 1 Hospital Universitario Dr. José Eleuterio González, Monterrey, Nuevo León, Mexico; 2 Hospital Civil de Guadalajara e Instituto de Patología Infecciosa, Guadalajara, Jalisco, Mexico; 3 Instituto Nacional de Ciencias Médicas y Nutrición Salvador Zubirán, Ciudad de México, Mexico; 4 Hospital del Niño y del Adolescente Morelense, Cuernavaca, Morelos, Mexico; 5 Instituto Nacional de Rehabilitación Luis Guillermo Ibarra Ibarra, Ciudad de México, Mexico; 6 Hospital general Dr. Manuel Gea González, Ciudad de México, Mexico; 7 Instituto Nacional de Cancerología, Ciudad de México, Mexico; 8 Hospital Regional Universitario de los Servicios de Salud del Estado de Colima y Facultad de Medicina, Universidad de Colima, Colima, Colima, Mexico; 9 Hospital Ángeles Valle oriente, Monterrey, Nuevo León, Mexico; 10 Hospital general Dr. Raymundo Abarca Alarcón, Chilpancingo, Guerrero, Mexico; 11 Hospital General de Mexicali/Facultad de Medicina Mexicali UABC, Mexicali, Baja California, Mexico; 12 Swiss Hospital, Monterrey, Nuevo León, Mexico; 13 Hospital General de Zona n°1, Tapachula, Chiapas, Mexico; 14 Laboratorio Estatal de Salud Pública de Michoacán, Morelia, Michoacán, Mexico; 15 Hospital Infantil de Morelia, Morelia, Michoacán, Mexico; 16 Hospital de Especialidades Pediátricas de Chiapas, Tuxtla Gutiérrez, Chiapas, Mexico; 17 Hospital Clínica Nova, Monterrey, Nuevo León, Mexico; 18 Hospital General de Mérida Yucatán “Dr. Agustín O ‘Horan”, Mérida, Yucatán, Mexico; 19 Hospital Regional de Alta Especialidad Bicentenario de la Independencia, Tultitlán de Mariano Escobedo, Estado de México, Mexico; 20 Hospital Ángeles de Morelia, Morelia, Michoacán, Mexico; 21 Hospital para el Niño Toluca IMIEM, Toluca, Estado de México, Mexico; 22 Hospital Regional de Alta Especialidad del Bajío, León, Guanajuato, Mexico; 23 Hospital General Presidente Lázaro Cárdenas del Rio, Chihuahua, Chihuahua, Mexico; 24 Sanatorio La Luz, Morelia, Michoacán, Mexico; 25 Hospital de Alta Especialidad de Veracruz, Veracruz, Veracruz, Mexico; 26 Hospital para el Niño Poblano, Puebla, Puebla, Mexico; 27 Hospital Dr. Jesús Gilberto Gómez Maza, Tuxtla Gutiérrez, Chiapas, Mexico; 28 Centenario Hospital Miguel Hidalgo, Aguascalientes, Aguascalientes, Mexico; 29 Labortorio Dipromi, Morelia, Michoacán, Mexico; 30 Hospital General con Especialidades Juan María de Salvatierra, La Paz, Baja California Sur, Mexico; 31 Hospital Regional Delicias, Delicias, Chihuahua, Mexico; 32 Hospital de la Mujer de Ciudad Juárez, Ciudad Juárez, Chihuahua, Mexico; 33 Hospital de Especialidades Pediátrico de León, León, Guanajuato, Mexico; 34 Hospital Infantil de Especialidades de Juárez, Ciudad Juárez, Chihuahua, Mexico; 35 Laboratorio Jurisdiccional de Ciudad Juárez, Ciudad Juárez, Chihuahua, Mexico; 36 Hospital Ángeles de Chihuahua, Chihuahua, Chihuahua, Mexico; 37 Galenia Hospital, Cancún, Quintana Roo, Mexico; 38 Hospital Regional de Alta Especialidad Cd. Victoria, Ciudad Victoria, Tamaulipas, Mexico; 39 Hospital Materno Infantil Morelos de Chetumal, Chetumal, Quintana Roo, Mexico; 40 Hospital General de zona 21 Tepatitlán de Morelos, Tepatitlán de Morelos, Jalisco, Mexico; 41 Centro Universitario de Salud, UANL Pueblo Nuevo, Monterrey, Nuevo León, Mexico; 42 Centro Universitario de Salud, UANL Vicente Guerrero, Monterrey, Nuevo León, Mexico; 43 Laboratorios del Centro, Zamora, Michoacán, Mexico; 44 Laboratorio de Análisis Bioquímico Clínicos "Louis Pasteur", Toluca, Estado de México, Mexico; 45 Hospital General Regional 220, Toluca, Estado de México, Mexico; 46 Hospital General de Chetumal, Chetumal, Quintana Roo, Mexico; 47 Hospital Arada de la Parra, León, Guanajuato, Mexico; 48 Instituto Nacional de Salud Pública, Cuernavaca, Morelos, Mexico; Emory University School of Medicine, UNITED STATES

## Abstract

**Aim:**

We aimed to assess the resistance rates of antimicrobial-resistant, in bacterial pathogens of epidemiological importance in 47 Mexican centers.

**Material and methods:**

In this retrospective study, we included a stratified sample of 47 centers, covering 20 Mexican states. Selected isolates considered as potential causatives of disease collected over a 6-month period were included. Laboratories employed their usual methods to perform microbiological studies. The results were deposited into a database and analyzed with the WHONET 5.6 software.

**Results:**

In this 6-month study, a total of 22,943 strains were included. Regarding Gram-negatives, carbapenem resistance was detected in ≤ 3% in *Escherichia coli*, 12.5% in *Klebsiella* sp. and *Enterobacter* sp., and up to 40% in *Pseudomonas aeruginosa*; in the latter, the resistance rate for piperacillin-tazobactam (TZP) was as high as 19.1%. In *Acinetobacter* sp., resistance rates for cefepime, ciprofloxacin, meropenem, and TZP were higher than 50%. Regarding Gram-positives, methicillin resistance in *Staphylococcus aureus* (MRSA) was as high as 21.4%, and vancomycin (VAN) resistance reached up to 21% in *Enterococcus faecium*. *Acinetobacter* sp. presented the highest multidrug resistance (53%) followed by *Klebsiella* sp. (22.6%) and *E*. *coli* (19.4%).

**Conclusion:**

The multidrug resistance of *Acinetobacter* sp., *Klebsiella* sp. and *E*. *coli* and the carbapenem resistance in specific groups of enterobacteria deserve special attention in Mexico. Vancomycin-resistant enterococci (VRE) and MRSA are common in our hospitals. Our results present valuable information for the implementation of measures to control drug resistance.

## Introduction

The increasing prevalence of antimicrobial resistance is a significant cause of concern in the field of public health. This issue requires an international approach for its management, although national and local strategies are also necessary [1]. The World Health Organization (WHO) has recognized the importance of studying the emergence of drug-resistant pathogens and the need of control strategies [2].

Both global and regional surveillance of drug resistance is fundamental for the implementation of adequate infection control measures and disease management [3]. For this reason, some research groups from Mexico have reported the drug resistance rates of some bacterial pathogens, including *Enterobacter* sp., *Klebsiella pneumoniae*, *Acinetobacter baumannii*, *Pseudomonas aeruginosa*, *Staphylococcus aureus*, and *Enterococcus faecium* [4–7]; with species producing extended-spectrum beta-lactamases (ESBLs) or carbapenemases receiving special consideration [8–13].

Information generated in Mexico is provided from specific areas of the country–such as Jalisco, Mexico City, and Nuevo Leon, with little or lacking information from Chiapas, Guerrero, Veracruz, Baja California, Colima, Aguascalientes, Chihuahua, Yucatan, Quintana Roo, and other states available. Given the overwhelming global situation, the Mexican government published an agreement declaring the compulsory nature of the National Strategy of Action Against Resistance to Antimicrobials that establishes the objectives and main strategies in order to improve usage of antibiotics and combat antimicrobial resistance, which should be adopted with a gradual approach, in the next 5 to 10 years (http://dof.gob.mx/nota_detalle.php?codigo=5525043&fecha=05/06/2018).

To contribute to the growing knowledge of drug resistance in Mexico, the Network for the Research and Surveillance of Drug Resistance (*Red Temática de Investigación y Vigilancia de la Farmacorresistencia* in Spanish) was created, and as part of the work of this network, we have aimed to create a picture of the drug resistance of Gram-positives and Gram-negatives in Mexico through the participation of 47 laboratories from 20 states across Mexico.

## Materials and methods

### Participating centers and data collection

Data from laboratories of different types of hospitals such as number of beds, population treated, and other criteria, as well as from external laboratories was considered. They all were from different states of the country. A total of 47 centers were included: 39 hospital-based laboratories and 8 external laboratories.

Demographic data regarding the number of beds, intensive care unit (ICU) capacity, and days of hospital stay were gathered as well as data about the laboratory identification and susceptibility testing methods, including the automated system and software used.

Each laboratory identified the strains they recovered and performed their susceptibility tests using conventional methods. Forty-three laboratories used commercial microdilution systems: 23 used VITEK 2 (Biomérieux, Marcy l’ Etoile, France); 11 used the Phoenix Automated Microbiology System (Becton-Dickinson, Sparks, MD, USA); 6 used MicroScan WalkAway (Siemens Healthcare Diagnostics, West Sacramento, CA, USA); and 3 used Sensititre (TEK Diagnostic Systems Inc, Cleveland OH). Five laboratories used the Clinical and Laboratory Standards Institute (CLSI) disk diffusion susceptibility method.

Hospitals submitted their results into a database, which was then sent to the coordinating hospital (Hospital Universitario Dr. José Eleuterio González, in the state of Nuevo Leon), where the results were analyzed and validated using the laboratory-based WHONET 5.6 program from WHO Collaborating Centre for the Surveillance of Antibiotic Resistance. A clinical microbiologist cautiously reviewed all records. Duplicated isolates, i.e. more than one isolate per patient, were identified and omitted from the analysis. Discrepancies and atypical results were resolved with the representative from each hospital, and the corresponding database records were updated if necessary. The results were scored as susceptible, intermediate, or resistant according to CLSI criteria (2017) in all laboratories [14].

### Strains included and analysis of the data

Clinical isolates collected from January 1 to June 30 of 2018 were included. The survey instrument addressed the distribution of antimicrobial resistance of several pathogens, including *Escherichia coli*, *Klebsiella* sp., *Enterobacter* sp., *Salmonella* sp., *Shigella* sp., *A*. *baumannii*, *P*. *aeruginosa*, *Stenotrophomonas maltophilia*, *S*. *aureus* and *Enterococcus* sp., in clinical specimens such as urine, respiratory specimens (tracheal aspirate and bronchial lavage), blood, cerebrospinal fluid (CSF), and feces. Only pathogens with epidemiological relevance and with the result of more than 10 isolates to determine percentages of drug susceptibility were included.

From each hospital, all data collected during the 6-month period was deposited into the WHONET platform. The conversion of the text file to the WHONET format was done through the BacLink 2 tool, which was configured according to the automated equipment used with the standardized dictionary defined in this protocol. All WHONET files of each hospital were combined, performing the quality control of the structure of the WHONET database with the use of the validation template. Macros created for this purpose were used to facilitate the revision.

### Ethics Statement

The local ethics committee (Comité de Ética en Investigación del Hospital Civil de Guadalajara “Fray Antonio Alcalde,” Jalisco, Mexico) approved this study with reference number 129/17. Informed consent was waived by the ethics committee because no intervention was involved and no patients’ identifying information was included. The ethics committee of all participating institutions agreed with the present study.

## Results

### Characteristics of the participating laboratories

In this 6-month study, 47 laboratories reported data with 39 being hospital-based laboratories and 8 being external laboratories. Of the hospital-based laboratories, 32/39 (82%) were from public hospitals, and 7/39 (18%) were from private hospitals. Among external laboratories, two were public health laboratories, and seven were private.

The centers were distributed across 20 Mexican states, with 16 (41%) hospitals having <100 beds, 15 (38.5%) 100–199, and 8 (20.55%) ≥ 200 beds. The characteristics of the participating hospitals are listed in Table 1. One of the hospitals provided data from only the ICU, and a second hospital reported only the selected pathogens in the chosen specimens. A third laboratory reported only a 3-month period. All other centers reported the complete data of the 6-month period.

**Table 1:** The characteristics of the participant hospital centers (external laboratories were not included).

Center	Type	Hosp beds	ICU beds	Hospitalizations in 2017				Length of stay (days) 2017			2017				
				Total	Obs	ICU ad	ICU ped	Obs	ICU ad	ICU ped	NB	Surg proc	UC-days	Vent-days	CVC-days
*1*	Univ	≥ 200	46	27,991	10,021	918	1,258	22,952	7,198	12,207	NR	11703	23,341	9,381	124,891
*2*	Unv	≥ 200	85	32,706	4,141	899	312	11,548	5,510	2,031	NR	20,639	32,410	15,651	51,699
*3*	Ped	< 100	15	NR	0	0	NR	0	0	NR	NR	NR	NR	NR	NR
*4*	Spe	≥ 200	20	7,407	NR	213	NR	NR	19,576	0	0	5,623	8,538	1,716	4,653
*5*	Gen	100–199	5	12,146	3,148	384	932	157,400	19,200	2,796	620	5,970	15,291	7,740	36,975
*6*	Spe	100–199	6	7,010	NA	237	0	0	1,474	0	0	3,155	5,852	1,278	14,602
*7*	Univ	100–199	8	9,207	3,348	7,344	NR	4,644	87	3,985	2,788	6,234	NR	NR	NR
*8*	Spe	< 100	21	5,373	1,277	139	26	3,696	620	2,003	1,224	3,477	2,902	754	2,185
*9*	Gen	100–199	8	NR	NR	NR	NR	NR	NR	NR	NR	NR	NR	NR	NR
*10*	Spe	< 100	2	8,750	0	730	0	NR	NR	NR	NR	NR	NR	NR	NR
*11*	Gen	< 100	9	7,148	2,483	80	158	0	360	1,444	2,017	5,081	NR	NR	NR
*12*	Spe	234	14	4,775	0	NR	NR	NR	NR	0	NR	2,489	NR	NR	NR
*13*	Ped	< 100	6	8,635	0	0	416	0	0	1,152	NR	5,181	635	1,108	2,099
*14*	Ped	< 100	19	1,654	0	0	224	0	0	4,231	NR	2770	1,796	2,903	12,421
*15*	Spe	< 100	4	NR	NR	NR	0	NR	NR	NR	368	2,134	1,356	53	818
*16*	Gen	≥ 200	24	NR	NR	468	259	NR	3,067	2,244	NR	NR	NR	NR	NR
*17*	Spe	≥ 200	8	9,709	3,257	313	220	8,583	1,522	282	1,327	9,300	7,528	3,254	6,168
*18*	Spe	< 100	6	5,267	390	19	2	722	107	15	318	1,762	1,278	125	513
*19*	M&Ch	100–199	30	4,646	0	0	300	0	0	2,399	0	4,041	2,976	3,910	6,463
*20*	Spe	100–199	29	7,459	0	714	290	0	2,452	1,630	0	5,762	9,410	7,432	16,378
*21*	Gen	100–199	19	5,452	948	268	109	NR	NR	NR	378	2,311	3,832	675	3,093
*22*	Gen	< 100	3	1,635	63	30	0	189	120	0	63	763	198	13	34
*23*	Esp	≥ 200	NR	NR	NR	NR	NR	NR	NR	NR	NR	NR	NR	NR	NR
*24*	Ped	< 100	17	3,292	NR	NR	NR	0	0	1,782	NR	4,067	NR	1,513	12,300
*25*	Gen	100–199	34	10,668	0	205	NR	0	1,845	NR	0	2,520	NR	NR	NR
*26*	Pu	100–199	21	14,568	0	445	548	0	1,685	2,513	3	7,607	7,311	3,100	11,162
*27*	Spe	100–199	18	9,106	2,277	221	285	4,607	1,059	2,281	NR	2,920	4,003	2,205	5,377
*28*	Spe	< 100	4	4,605	1,947	103	92	3,116	258	729	NR	NR	NR	NR	NR
*29*	M&Ch	100–199	22	11,089	NR	95	NR	NR	NR	NR	NR	NR	NR	NR	NR
*30*	Ped	< 100	17	2,728	0	0	469	0	0	5,782	0	2,144	NR	563	441
*31*	Ped	< 100	20	2,908	0	0	2,908	0	0	253	0	1,559	89	163	387
*32*	Spe	100–199	16	NR	NR	NR	NR	NR	NR	NR	NR	NR	NR	NR	NR
*33*	Spe	< 100	4	2,822	482	80	NA	926	444	NA	499	1,970	2,190	369	976
*34*	Spe	100–199	NR	NR	NR	NR	NR	NR	NR	NR	NR	NR	NR	NR	NR
*35*	M&Ch	< 100	0	3,551	3,389	0	0	4,103	0	0	3,200	1,455	49	644	1,761
*36*	Gen	< 100	9	7,148	2,483	80	158	NR	360	1,444	2,017	5,081	NR	NR	NR
*37*	Gen	≥ 200	20	14,845	0	183	152	0	4,524	2,520	0	8157	16,937	7013	13,119
*38*	Gen	100–199	10	NR	NR	NR	NR	NR	NR	NR	NR	NR	NR	NR	NR
*39*	Spe	100–199	6	7,378	NR	NR	NR	NR	NR	NR	NR	1,749	957	1,788	NR

NR: not reported, Univ: university, Gen: general, Spe: specialties, M&Ch: mother and child, Ped: pediatrics, Ad: adults, Surg Proc: surgical procedures, Vent-days: days of ventilator usage, CVC-days: days of central venous catheter usage, UC-days: days of urine catheter usage, and NB: newborn.

### Prevalence of resistance to antimicrobial agents

A total of 22,943 strains from all laboratories were included. For the evaluation of drug resistance, we selected antibiotics to report according to CLSI guidelines and the information available from centers. The frequency of antimicrobial resistance of Gram-negatives and Gram-positives in all centers is shown in Tables 2 and 3, respectively. In the initial analysis, drug resistance rates were calculated for all specimens–where the species may be considered as a causative agent including respiratory, urine, blood and others such as abscess, biopsies, among others–and for specific specimens including respiratory specimens (only tracheal aspirate and bronchial lavage), urine (any collection), blood, CSF, and feces. Regarding Gram-negatives, *E*. *coli* showed a carbapenem resistance of ≤ 3%, with amikacin exhibiting good activity (≤ 2% resistance). Third- and fourth-generation cephalosporins’s resistance was higher than 50%, and resistance rates for trimethoprim-sulfamethoxazole (SXT) were higher than 60%. In *Klebsiella* sp., carbapenem resistance reached as high as 12.5% for respiratory specimens. Similar results to that of *E*. *coli* were observed for third- and fourth-generation cephalosporins. In *Enterobacter* sp., carbapenem resistance was similar to that of *Klebsiella* sp., with lower resistance for third-generation cephalosporins (up to 44.8%). In *P*. *aeruginosa*, up to 40%, carbapenem resistance was detected, and a resistance rate as high as 19.1% was detected for piperacillin-tazobactam (TZP) in specimens collected from urine. In *A*. *baumannii*, the resistance rates for cefepime (FEP), ciprofloxacin (CIP), meropenem (MEM), and TZP were higher than 50%, with only tobramycin (TOB) and gentamicin showing resistance rates lower than 44% in the specimens evaluated.

**Table 2:** The rates of antimicrobial resistance in percentages for selected Gram-negative pathogens at 47 centers according to specimens.

Genus/species	Specimen (n)	AMK	AMC	AMP	SAM	AZM	CFZ	FEP	FOX	CRO	CIP	ETP	GEN	IMP	LVX	MEM	NIT	TZP	TGC	TOB	SXT
*E*. *coli*	All (11,676)	1.8	39.8	82.2	46.8	52.9	54.4	52.4	47.6	50.9	59.0	1.7	36.7	1.7	59.4	0.8	5.0	8.9	0.2	26.0	62.1
	URI (6,592)	1.8	41.2	81.7	46.5	ND	52.7	51.3	54.9	49.0	61.6	1.2	35.5	1.2	64.6	0.5	5.7	8.3	ND	25.6	61.5
	BLO (274)	0.0	30.9	92.3	60.0	64.6	69.0	68.9	72.4	68.4	62.8	2.0	42.3	3.0	66.2	1.5	4.5	14.9	0.0	24.7	73.2
	CSF (20)	0.0	ND	ND	60.0	73.3	73.3	80.0	ND	80.0	50.0	0.0	75.0	0.0	ND	0.0	0.0	20.0	0.0	40.0	30.0
*Klebsiella* sp.	All (3,334)	3.9	37.9	ND	55.0	55.5	61.1	52.5	52.7	53.7	31.1	6.5	43.5	6.9	26.4	5.6	19.9	13.5	1.0	41.1	56.8
	RES (299)	3.9	20.7	ND	62.5	54.1	62.7	56.8	48.3	56.8	29.0	5.9	46.6	12.5	39.5	6.5	15.4	15.0	0.8	45.9	60.4
	URI (1,052)	3.9	41.0	ND	55.5	56.6	62.6	52.8	58.9	52.9	34.1	5.4	41.5	2.5	24.4	4.6	26.6	14.7	ND	35.6	57.2
	BLO (166)	1.0	27.8	ND	68.1	74.4	79.0	70.0	59.1	70.9	45.9	3.6	63.1	1.8	48.0	3.6	21.0	7.3	0.0	62.6	68.2
	CSF (21)	31.6	ND	ND	89.5	100.0	100.0	100.0	ND	100.0	63.2	10.5	90.5	ND	ND	10.5	0.0	26.3	0.0	100.0	73.7
*Enterobacter* sp.	All (1,334)	6.1	ND	ND	ND	35.1	91.7	19.5	ND	42.1	13.8	11.8	15.8	ND	ND	9.9	15.2	26.7	0.6	14.2	26.3
	URI (401)	4.7	ND	ND	ND	ND	ND	22.1	ND	44.8	18.9	10.8	17.1	ND	ND	8.8	25.6	27.5	1.2	10.8	30.1
	BLO (67)	7.1	ND	ND	ND	35.0	92.0	16.7	ND	40.0	13.3	6.7	6.5	ND	ND	3.3	10.0	26.7	0.0	18.2	16.7
*P*. *aeruginosa*	All (1,995)	17.3	ND	ND	ND	12.8	ND	17.5	ND	ND	18.6	ND	16.7	29.7	22.4	27.8	ND	14.8	ND	17.5	ND
	RES (370)	14.9	ND	ND	ND	15.4	ND	7.2	ND	ND	13.2	ND	14.6	27.0	11.3	32.7	ND	7.5	ND	17.6	ND
	URI (342)	30.1	ND	ND	ND	ND	ND	28.4	ND	ND	35.9	ND	31.1	ND	ND	31.3	ND	19.1	ND	31.5	ND
	BLO (197)	7.7	ND	ND	ND	4.5	ND	17.4	ND	ND	11.36	ND	5.8	30.0	15.2	20.3	ND	8.8	ND	3.5	ND
	CSF (28)	10.0	ND	ND	ND	ND	ND	0.0	ND	ND	0.0	ND	0.0	0.0	0.0	40.0	ND	0.0	ND	11.1	ND
*Acinetobacter* sp	All (861)	ND	ND	ND	53.2	ND	ND	80.3	ND	ND	82.3	ND	42.5	ND	ND	79.6	ND	73.7	ND	37.4	ND
	RES (316)	ND	ND	ND	54.0	ND	ND	90.5	ND	ND	90.4	ND	43.5	ND	ND	86.4	ND	86.9	ND	36.0	ND
	URI (93)	ND	ND	ND	53.8	ND	ND	78.6	ND	ND	83.9	ND	42.9	ND	ND	82.1	ND	69.2	ND	40.7	ND
	BLO (58)	ND	ND	ND	17.1	ND	ND	54.1	ND	ND	50.0	ND	7.9	ND	ND	52.6	ND	60.0	ND	9.1	ND
	CSF (18)	ND	ND	ND	44.4	ND	ND	81.2	ND	ND	81.2	ND	16.7	ND	ND	81.2	ND	80.0	ND	6.2	ND
*Salmonella* sp.	All (71)	ND	ND	28.0	ND	ND	ND	ND	ND	ND	27.7	ND	ND	ND	ND	ND	ND	ND	ND	ND	17.4
	FEC (41)	ND	ND	11.8	ND	ND	ND	ND	ND	ND	6.2	ND	ND	ND	ND	ND	ND	ND	ND	ND	13.3
*S*. *typhi*	All (10)	ND	ND	0.0	ND	ND	ND	ND	ND	0.0	20.0	ND	ND	ND	ND	ND	ND	ND	ND	ND	0.0
*Shigella* sp.	FEC (28)	ND	ND	25.0	ND	ND	ND	ND	ND	ND	25.0	ND	ND	ND	25.0	ND	ND	ND	ND	ND	37.5
*S*. *maltophilia*	RES (60)	ND	ND	ND	ND	ND	ND	ND	ND	ND	ND	ND	ND	ND	11.1	ND	ND	ND	ND	ND	8.8

RES: respiratory specimens (tracheal aspirate and bronchial washing), URI: urine, BLO: blood; CSF: cerebrospinal fluid, FEC: feces, ND: not determined, AMK: amikacin, AMC: amoxicillin-clavulanic acid, AMP: ampicillin, SAM: ampicillin-sulbactam, AZT: aztreonam, CFZ: cefazolin, FEP: cefepime, FOX: cefoxitin, CRO: ceftriaxone, CIP: ciprofloxacin, ETP: ertapenem, GEN: gentamicin, IMP: imipenem, LVX: levofloxacin, MEM: meropenem, NIT: nitrofurantoin, TZP: piperacillin-tazobactam, TGC: tigecycline, TOB: tobramycin, and SXT: trimethoprim-sulfamethoxazole.

**Table 3:** The rates of antimicrobial resistance in percentages for selected Gram-positive pathogens at 47 centers according to specimens.

Pathogen	Specimen (n)	AMP	FOX	CPT	CHL	CIP	CLI	ERY	GEN	LVX	LZD	MIN	MXF	NIT	OXA	PEN	SYN	TEC	TET	SXT	VAN
*S*. *aureus*	All (2,646)	ND	25.0	0.0	0.9	26.3	32.3	32.9	8.4	27.5	0.7	1.8	23.1	0.6	23.1	90.3	1.7	1.8	4.8	4.7	0.0
	RES (144)	ND	21.4	ND	ND	22.5	27.5	27.1	6.6	22.1	1.1	ND	20.2	0.0	20.2	84.8	0.0	ND	6.2	3.0	0.0
	URI (91)	ND	9.1	ND	ND	21.4	ND	20.0	ND	20.0	0.0	ND	16.7	3.7	14.3	77.8	0.0	ND	ND	7.4	0.0
	BLO (293)	ND	16.7	0.0	10.0	24.4	28.8	26.9	10.7	25.4	1.0	0.0	19.7	1.6	18.4	95.6	5.3	10.0	4.8	8.7	0.0
*E*. *faecalis*	All (892)	6.1	ND	ND	ND	35.5	ND	58.6	ND	35.8	7.3	ND	ND	3.0	ND	16.6	84.4	ND	80.9	ND	4.3
	URI (270)	7.4	ND	ND	ND	42.4	ND	63.2	ND	41.5	7.5	ND	ND	ND	ND	20.0	86.5	ND	87.6	ND	5.2
	BLO (30)	3.6	ND	ND	ND	20.0	ND	38.1	ND	20.0	3.6	ND	ND	ND	ND	14.3	94.4	ND	83.3	ND	3.6
*E*. *faecium*	All (124)	73.2	ND	ND	ND	60.8	ND	80.8	ND	58.4	2.4	ND	ND	17.1	ND	74.1	9.7	ND	47.7	ND	20.7
	URI (38)	80.6	ND	ND	ND	77.8	ND	93.1	ND	72.2	2.8	ND	ND	11.4	ND	89.3	11.5	ND	57.7	ND	25.0

RES: respiratory (tracheal aspirate and bronchial washing), URI: urine, BLO: blood, ND: not determined, AMP: ampicillin, FOX: cefoxitin, CPT: ceftaroline, CHL: chloramphenicol, CIP: ciprofloxacin, CLI: clindamycin, ERY: erythromycin, GEN: gentamicin, LVX: levofloxacin, LZD: linezolid, MIN: minocycline, MXF: moxifloxacin, NIT: nitrofurantoin, OXA: oxacillin, PEN: penicillin, SYN: quinupristin-dalfopristin, TEC: teicoplanin, TET: tetracycline, SXT: trimethoprim-sulfamethoxazole, and VAN: vancomycin.

In *Salmonella* sp., resistance rates were 27.7% and 17.4% for SXT and CIP, respectively. *Shigella* sp. revealed resistance rates of ≥ 25% for ampicillin (AMP), CIP, and SXT. In *S*. *maltophilia*, resistance to levofloxacin (LVX) and SXT was around 10%.

Regarding Gram-positives, methicillin resistance in *S*. *aureus* (MRSA) was as high as 21.4% for respiratory specimens, though good activity was detected for SXT (3.0–8.7%). Vancomycin resistance in *Enterococcus* sp. (VRE) ranged from 3.6% to 5.2% in *Enterococcus faecalis*, with good activity for AMP (from 3.6% to 7.4%); resistance to LZD was 7.3% for all specimens. Also, as expected, higher and important resistance rates to vancomycin were found in *E*. *faecium*, which were 21% for all specimens.

Strains were classified as multidrug-resistant (MDR), possible extensively drug-resistant (XDR), true XDR or possible pandrug-resistant (PDR) [15]. *A*. *baumannii* presented the highest MDR rate (53%) followed by *Klebsiella* sp. (22.6%) and *E*. *coli* (19.4%) (Table 4). Interestingly, 43.2% of *A*. *baumannii* isolates showed to be possible XDR, 8.8% true XDR and 38.8% possible PDR.

**Table 4:** Distribution of MDR, PDR and XDR among Gram negatives in all specimens.

Microorganism (n)	MDR n (%)	Possible XDR n (%)	True XDR n (%)	Possible PDR n (%)
*Acinetobacter* sp. (861)	459 (53.0)	372 (43.2)	76 (8.8)	334 (38.8)
*Klebsiella* sp. (3334)	752 (22.6)	ND	ND	ND
*E*. *coli* (11676)	2261 (19.4)	942 (8.1)	0 (0)	5 (0.04)
*Enterobacter* sp. (1334)	159 (11.9)	ND	ND	ND
*P*. *aeruginosa* (1995)	175 (8.8)	165 (8.3)	3 (0.2)	87 (4.4)

**ND**: no data. Only the species in which the calculations were possible according to the available information were included.

## Discussion

Antimicrobial resistance is a concerning problem worldwide with resistance rates differing among countries. To adequately address this decisive issue, data on drug resistance trends are fundamental. In this study, we report the frequencies of drug resistance of the most representative bacterial pathogens using the routine results from 47 centers in Mexico.

Although existing antimicrobial resistance surveillances in some hospitals in Mexico is in progress, these programs have significant limitations. For example, reports of drug resistance are overrepresented by larger teaching hospitals [16–20], and there is less available information about smaller, non-teaching hospitals and external laboratories. Furthermore, valuable data generated by projects sponsored by pharmaceuticals such as SENTRY Antimicrobial Surveillance Program [21, 22], Tigecycline Evaluation and Surveillance Trial (TEST, not currently active) [23], and the Study for Monitoring Antimicrobial Resistance Trends (SMART) [17], is available, although these studies are focused on one or few organisms and a limited set of tested antibiotics.

In the results of the current study, amikacin demonstrated a low resistance rate against *E*. *coli* (lower than 2%) suggesting it remains a valuable option for the management of urinary tract infections (UTIs) and it also maintains activity against *P*. *aeruginosa* isolated from blood cultures (lower than 10%). These data render this antibiotic an effective therapeutic alternative if used in combination with other drugs. However, it should be considered that aminoglycosides are one of the causes of drug-induced nephrotoxicity and ototoxicity [24]; thus, close patient monitoring is required, and other therapeutic alternatives such as fosfomycin and nitrofurantoin should be considered.

The potential production of ESBLs detected by resistance to aztreonam (AZM), FEP and ceftriaxone in enterobacteria is alarming, at around 50%. The first ESBLs were detected in Mexico nearly 20 years ago [25], and now the country is overwhelmed by the presence of bacteria carrying these enzymes. It is well known that ESBL production reduces alternative therapeutics for all infections, and this situation is worsened by the high resistance observed to fluoroquinolones–up to 63.2% for CIP and 66.2% for LVX in our study. The combined resistance to cephalosporins and fluoroquinolones in *E*. *coli* may be related to the presence of the sequence type (ST) 131 because in this particular clonal group the resistance reported to these antibiotic groups is higher than 65% [26]. The circulation of *E*. *coli* ST131 has been reported in Mexico since 2011 [27, 28]. Interestingly, the resistance detected to SXT in *E*. *coli* was high: 61.5% for urine isolates and 62.1% for all isolates. Thus, SXT should be excluded from empirical UTI treatment.

In *Salmonella* sp. the resistance rate to CIP was 17.4%. In contrast, a recent report that included the analysis of a frozen collection of both animal and human isolates (35 from the latter group), exhibited no resistance to this drug [29].

In our study, the analysis of 28 isolates of *Shigella* sp. revealed resistance rates of ≥ 25% to AMP, CIP, and SXT. A previous report demonstrated resistance to AMP and SXT of 40% and 58%, respectively, and no resistance was detected to CIP [30].

Most of carbapenem resistance rates in *Enterobacteriaceae* were lower than 10%. There are some reports of carbapenem resistance in enterobacteria, including the carbapenemase production in Mexico, especially for *K*. *pneumoniae* and *E*. *coli* [12, 18]. In this study, we confirmed the carbapenem resistance of enterobacteria in Mexico.

In *Acinetobacter* sp., the generalized drug resistance detected is alarming because almost no therapeutic options are currently available. High multidrug resistance has been reported in Mexico in several focalized studies [20, 31, 32]. Drug resistance in *Acinetobacter* sp. should be considered a priority in Mexico because an attributable mortality rate higher than 25% has been reported for infections associated to this bacterial species [33]. Fighting against this infection should include all known measures for control of hospital infections such as hand sanitization, isolation of patients, and antimicrobial stewardship.

Regarding Gram-positives, we detected vancomycin (VAN) resistance higher than 20% in *E*. *faecium*, and in *E*. *faecalis*, we identified a LZD resistance of 7.3%. Furthermore, we observed a 0.7% resistance to LZD in *S*. *aureus*. Our study is based on routine laboratory results, and no confirmation of rare phenotypes was performed, with some exceptions. Resistance to LZD in enterococci was confirmed in two centers.

This work has some significant limitations. First, we did not include the results of colistin in Gram-negatives as only the laboratories that used the Phoenix machine reported the results of this antibiotic. Second, some valuable information was incomplete, such as the wards where the patients were hospitalized in and the gender and age of the patients; therefore, we decided to analyze the variables for which we had complete information. Last, we experienced the significant disadvantages of the use of routine results including the different methods of antimicrobial susceptibility testing performed in each laboratory. In our work, most laboratories used CLSI-recommended methodologies, and with the use of WHONET software, we were able to homogenize the interpretation values to the 2017 document. However, quality control and corroboration of resistant results were not used for this initial report.

During this study, centers were trained about the use of WHONET software and received comments on the actions needed to improve surveillance; therefore, all centers plan to continue active surveillance with the use of this instrument, including all data.

Our study does not pretend to be a surveillance study because the study period was short (6 months), but to reflect a unique snapshot of the drug resistance in Mexico with information from 20 states that would be useful to define better strategies to control drug resistance.

Hospital antibiotic restriction is an effective measure to control antibiotic resistance and according to this, hospitals should eliminate or at least restrict antibiotics in which high resistance is observed and replace them with equivalent antibiotics with low resistance potential. According to our results, antibiotics in which high resistance was observed should be eliminated (e.g. CRO and CIP), and should be replaced with antibiotics which exhibeted low-resistance (e.g. AMK, or carbapenems in some species). Furthermore, VAN use should be restricted, and options such as LNZ should be considered. Antibiotic resistance is a worldwide concern and information generated in this study will be used to define strategies to better control resistance, develop more antimicrobial stewardship programs in Mexico, support the national strategies to combat antimicrobial resistance and promote the prudent use of antibiotics.

In this study, we included 7 out of the 12 pathogens the WHO published as antibiotic-resistant priority pathogens (http://www.who.int/news-room/detail/27-02-2017-who-publishes-list-of-bacteria-for-which-new-antibiotics-are-urgently-needed): carbapenem-resistant *A*. *baumannii* and *P*. *aeruginosa*, carbapenem-resistant ESBL-producing *Enterobacteriaceae*, VAN-resistant *E*. *faecium*, MRSA, and fluoroquinolone-resistant *Salmonella* and *Shigella* sp. We did not include *Helicobacter pylori*, *Campylobacter* spp., *Neisseria gonorrhoeae*, *Streptococcus pneumoniae*, nor *Haemophilus influenzae*, because few information was available from centers.

In conclusion, the use of routine antimicrobial susceptibility results from the laboratories allowed us to produce a 6-month picture of the drug resistance of most bacterial species of epidemiological importance. The multidrug resistance of *Acinetobacter* sp., *Klebsiella* sp. and *E*. *coli* and the carbapenem resistance in specific groups of enterobacteria deserve special attention. VRE and MRSA are common in our hospitals. Our results present valuable information for the implementation of measures to control drug resistance.

## Supporting information

S1 Table Authors and participating centers. (PDF)

S2 Table Members of the Invifar group not included in the author list. (DOCX)
