# Supplementary material for: A snapshot of antimicrobial resistance in Mexico. Results from 47 centers from 20 states during a six-month period
Source: PLoS One. 2019 Mar 26;14(3):e0209865. doi: 10.1371/journal.pone.0209865 (PMC6435111; doi:10.1371/journal.pone.0209865)
Supplement: S1 Table — (PDF) [file pone.0209865.s001.pdf]

S1 Table. Authors and participating centers

No Center	Author	Hospital or center
1	Elvira Garza-González*	Hospital Universitario Dr. José Eleuterio González, Monterrey, Nuevo León, Mexico.
2	Rayo Morfín-Otero	Hospital Civil de Guadalajara e instituto de patología infecciosa, Guadalajara, Jalisco, Mexico.
	Soraya Mendoza-Olazarán	Hospital Universitario Dr. José Eleuterio González, Monterrey, Nuevo León, Mexico.
	Paola Bocanegra-Ibarias	Hospital Universitario Dr. José Eleuterio González, Monterrey, Nuevo León, Mexico.
	Samantha Flores-Treviño	Hospital Universitario Dr. José Eleuterio González, Monterrey, Nuevo León, Mexico.
	Eduardo Rodríguez-Noriega	Hospital Civil de Guadalajara e instituto de patología infecciosa, Guadalajara, Jalisco, Mexico.
3	Alfredo Ponce-de-León	Instituto Nacional de Ciencias Médicas y Nutrición Salvador Zubirán, CDMX, Mexico.
4	Domingo Sánchez-Francia	Hospital del Niño y del Adolescente morelense, Cuernavaca, Morelos, Mexico.
5	Rafael Franco-Cendejas	Instituto Nacional de Rehabilitación Luis Guillermo Ibarra Ibarra, CDMX, Mexico.
6	Sara Arroyo-Escalante	Hospital General Dr. Manuel Gea González, CDMX, Mexico.
7	Consuelo Velázquez-Acosta	Instituto Nacional de Cancerología, CDMX, Mexico.
8	Fabián Rojas-Larios	Hospital Regional Universitario de los Servicios de Salud del Estado de Colima y Facultad de Medicina, Universidad de Colima, Colima, Colima, Mexico.
9	Luis J. Quintanilla	Hospital Ángeles Valle Oriente, Monterrey, Nuevo León, Mexico.
10	Joyarit Y. Maldonado-Anicacio	Hospital General Dr. Raymundo Abarca Alarcón, Chilpancingo, Guerrero, Mexico.
11	Rafael Martínez-Miranda	Hospital General de Mexicali/Facultad de Medicina Mexicali UABC, Mexicali, Baja California, Mexico.
12	Heidy L. Ostos-Cantú	Swiss Hospital, Monterrey, Nuevo León, Mexico.
13	Abraham Gómez-Choel	Hospital General de Zona No.1, Tapachula, Chiapas, Mexico.
14	Juan L. Jaime-Sánchez	Laboratorio Estatal de Salud Pública de Michoacán, Morelia, Michoacán, Mexico.
15	Laura K. Avilés-Benítez	Hospital Infantil de Morelia, Morelia, Michoacán, Mexico.
16	José M. Feliciano-Guzmán	Hospital de especialidades pediátricas de Chiapas, Tuxtla Gutiérrez, Chiapas, Mexico.
17	Cynthia D. Peña-López	Hospital Clínica Nova, Monterrey, Nuevo León, Mexico.
18	Carlos A. Couoh-May	Hospital general de Mérida Yucatán “Dr. Agustín O ‘Horan”, Mérida, Yucatán, Mexico.
19	Norma Alavez-Ramírez	Hospital Regional de alta especialidad Bicentenario de la independencia, Tultitlán de Mariano Escobedo, Estado de México, Mexico.
20	Elda G. Vázquez -Narváez	Hospital Ángeles de Morelia, Morelia, Michoacán, Mexico.
21	Joaquín Rincón-Zuno	Hospital para el Niño Toluca IMIEM, Toluca, Estado de México, Mexico.
22	Raúl Rivera-Garay	Hospital Regional de alta Especialidad del Bajío, León, Guanajuato, Mexico.
23	Aurelio Galindo-Espinoza	Hospital General presidente Lázaro Cárdenas del Rio, Chihuahua, Chihuahua, Mexico.
24	Andrés Martínez-Ramírez	Sanatorio La luz, Morelia, Michoacán, Mexico.
25	Javier P. Mora	Hospital de Alta Especialidad de Veracruz, Veracruz, Veracruz, Mexico.
26	Reyna E. Corte- Rojas	Hospital para el niño poblano, Puebla, Puebla, Mexico.
27	Ismelda López-Ovilla	Hospital Dr. Jesús Gilberto Gómez Maza, Tuxtla Gutiérrez, Chiapas, Mexico.
28	Víctor A. Monroy-Colin	Centenario Hospital Miguel Hidalgo, Aguascalientes, Aguascalientes, Mexico.
29	Juan M. Barajas-Magallón	Laboratorio Dipromi, Morelia, Michoacán, Mexico.
30	Cecilia T. Morales-De-la-Peña	Hospital General con Especialidades Juan María de Salvatierra, La Paz, Baja California Sur, Mexico.

31	Efrén Aguirre-Burciaga	Hospital Regional Delicias, Delicias, Chihuahua, Mexico.
32	Mabel Coronado-Ramírez	Hospital de la Mujer de ciudad Juárez, Ciudad Juárez, Chihuahua, Mexico.
33	Alina A. Rosales-García	Hospital de Especialidades Pediátrico de León, León, Guanajuato, Mexico.
34	María de J. Ayala-Tarín	Hospital Infantil de especialidades de Juárez, Ciudad Juárez, Chihuahua, Mexico.
35	Silvia Sida-Rodríguez	Laboratorio Jurisdiccional de Ciudad Juárez, Ciudad Juárez, Chihuahua, Mexico.
36	Bertha A. Pérez-Vega	Hospital Ángeles de Chihuahua, Chihuahua, Chihuahua, Mexico.
37	América Navarro-Rodríguez	Galenia Hospital, Cancún, Quintana Roo, Mexico.
38	Gloria E. Juárez-Velázquez	Hospital Regional de alta especialidad Cd. Victoria, Ciudad Victoria, Tamaulipas, Mexico.
39	Carlos Miguel Cetina-Umaña	Hospital Materno Infantil Morelos de Chetumal, Chetumal, Quintana Roo, Mexico.
40	Juan P. Mena-Ramírez	Hospital General de Zona Tepatitlán de Morelos, Tepatitlán de Morelos, Jalisco, Mexico.
41, 42	Jorge Canizales-Oviedo,	Centro Universitario de Salud, UANL Pueblo Nuevo, Monterrey, Nuevo León, Mexico.<br>Centro Universitario de Salud, UANL Vicente Guerrero, Monterrey, Nuevo León, Mexico.
43	Martha Irene Moreno-Méndez	Laboratorios del Centro, Zamora, Michoacán, Mexico.
44	Daniel Romero-Romero	Laboratorio de Análisis Bioquímico Clínicos "Louis Pasteur", Toluca, Estado de México, Mexico.
45	Alejandra Arévalo-Mejía	Hospital General regional. Toluca, Estado de México, Mexico.
46	Dulce Isabel Cobos-Canul	Hospital General de Chetumal, Chetumal, Quintana Roo, Mexico.
47	Gilberto Aguilar-Orozco	Hospital Arada de la Parra, León, Guanajuato, Mexico.
48	Jesús Silva-Sánchez	Instituto Nacional de Salud Pública, Cuernavaca, Morelos, Mexico.
1	Adrián Camacho-Ortiz	Hospital Universitario Dr. José Eleuterio González, Monterrey, Nuevo León, Mexico.
