# Supplementary material for: A snapshot of antimicrobial resistance in Mexico. Results from 47 centers from 20 states during a six-month period
Source: PLoS One. 2019 Mar 26;14(3):e0209865. doi: 10.1371/journal.pone.0209865 (PMC6435111; doi:10.1371/journal.pone.0209865)
Supplement: S2 Table — (DOCX) [file pone.0209865.s002.docx]

| CENTER | MEMBER |
| --- | --- |
| HOSPITAL CIVIL DE GUADALAJARA E INSTITUTO DE PATOLOGIA INFECCIOSA | ADOLFO GOMEZ QUIROZ |
| HOSPITAL CIVIL DE GUADALAJARA E INSTITUTO DE PATOLOGIA INFECCIOSA | ESTEBAN GONZÁLEZ DÍAZ |
| HOSPITAL CIVIL DE GUADALAJARA E INSTITUTO DE PATOLOGIA INFECCIOSA | GERARDO LEÓN GARNICA |
| HOSPITAL CIVIL DE GUADALAJARA E INSTITUTO DE PATOLOGIA INFECCIOSA | HÉCTOR RAÚL PÉREZ GÓMEZ |
| HOSPITAL CIVIL DE GUADALAJARA E INSTITUTO DE PATOLOGIA INFECCIOSA | JULIA RITA HEREDIA CERVANTES |
| INSTITUTO NACIONAL DE CIENCIAS MÉDICAS Y NUTRICIÓN SALVADOR ZUBIRÁN | ANA LILIA ROLÓN MONTESDEOCA |
| INSTITUTO NACIONAL DE CIENCIAS MÉDICAS Y NUTRICIÓN SALVADOR ZUBIRÁN | FERNANDO TUZ DZIB |
| INSTITUTO NACIONAL DE REHABILITACIÓN LUIS GUILLERMO IBARRA IBARRA | CLAUDIA ADRIANA COLÍN CASTRO |
| INSTITUTO NACIONAL DE REHABILITACIÓN LUIS GUILLERMO IBARRA IBARRA | LUIS ESAÚ LÓPEZ JÁCOME |
| INSTITUTO NACIONAL DE REHABILITACIÓN LUIS GUILLERMO IBARRA IBARRA | MELISSA HERNÁNDEZ DURÁN |
| HOSPITAL GENERAL DR. MANUEL GEA GONZALEZ | PATRICIA RODRÍGUEZ ZULUETA |
| INSTITUTO NACIONAL DE CANCEROLOGIA | DIANA VILAR COMPTE |
| INSTITUTO NACIONAL DE CANCEROLOGIA | PATRICIA CORNEJO JUAREZ |
| HOSPITAL REGIONAL UNIVERSITARIO DE LOS SERVICIOS DE SALUD DEL ESTADO DE COLIMA Y FACULTAD DE MEDICINA, UNIVERSIDAD DE COLIMA | FRANCISCO ESPINOZA GÓMEZ |
| HOSPITAL REGIONAL UNIVERSITARIO DE LOS SERVICIOS DE SALUD DEL ESTADO DE COLIMA Y FACULTAD DE MEDICINA, UNIVERSIDAD DE COLIMA | CARLOS MOISES HERNANDEZ SUAREZ |
| HOSPITAL REGIONAL UNIVERSITARIO DE LOS SERVICIOS DE SALUD DEL ESTADO DE COLIMA Y FACULTAD DE MEDICINA, UNIVERSIDAD DE COLIMA | IVÁN DELGADO DELGADO ENCISO |
| HOSPITAL REGIONAL UNIVERSITARIO DE LOS SERVICIOS DE SALUD DEL ESTADO DE COLIMA Y FACULTAD DE MEDICINA, UNIVERSIDAD DE COLIMA | OSCAR A. NEWTON SÁNCHEZ |
| HOSPITAL ANGELES VALLE ORIENTE | LOURDES GARCÍA MENDOZA |
| HOSPITAL ANGELES VALLE ORIENTE | NORA PATRICIA MUÑIZ MOLINA |
| HOSPITAL ANGELES VALLE ORIENTE | PABLO DANIEL TREVIÑO VALDÉZ |
| ESCUELA SUPERIOR DE CIENCIAS NATURALES Y HOSPITAL GENERAL DR RAYMUNDO ABARCA. DR. RAYMUNDO ABARCA ALARCÓN DE CHILPANCINGO, GRO. | NICOLAS ROGELIO ERIC RENDÓN BARLANDAS |
| ESCUELA SUPERIOR DE CIENCIAS NATURALES Y HOSPITAL GENERAL DR RAYMUNDO ABARCA. DR. RAYMUNDO ABARCA ALARCÓN DE CHILPANCINGO, GRO. | SANDRA QUINTANA PONCE |
| LABORATORIO DORADO HOSPITAL GENERAL DE MEXICALI | RODOLFO RUIZ LUJÁN |
| SWISSHOSPITAL | XIOMARA OLGA JIMENEZ DE LOPEZ |
| SWISSHOSPITAL | YAZMINE CAZARES CARRIZALES |
| SWISSHOSPITAL | ADRIANA LETICIA VALENCIA RAMOS |
| SWISSHOSPITAL | ERIC ANDRÉS GÓMEZ GONZÁLEZ |
| HOSPITAL GENERAL DE ZONA NO1 | ENRIQUE MARTINEZ RAMÍREZ |
| HOSPITAL GENERAL DE ZONA NO1 | ELENA VICTORIA CHOY CHANG |
| HOSPITAL GENERAL DE ZONA NO1 | JULIO CÉSAR JAN GÓMEZ |
| HOSPITAL GENERAL DE ZONA NO1 | MARÍA DE LOURDES MUÑOZ FIGUEROA |
| LABORATORIO ESTATAL DE SALUD PÚBLICA DE MICHOACÁN | DIANA CASARES OROZCO |
| LABORATORIO ESTATAL DE SALUD PÚBLICA DE MICHOACÁN | GLORIA ALICIA FIGUEROA AGUILAR |
| LABORATORIO ESTATAL DE SALUD PÚBLICA DE MICHOACÁN | GUADALUPE VIRIDIANA BARBOSA BOTELLO |
| LABORATORIO ESTATAL DE SALUD PÚBLICA DE MICHOACÁN | MARIA DE JESÚS GUEVARA LANDEROS |
| LABORATORIO ESTATAL DE SALUD PÚBLICA DE MICHOACÁN | MARIA VICENTA LUNA OLIVO |
| LABORATORIO ESTATAL DE SALUD PÚBLICA DE MICHOACÁN | MARIANA PALOMINO PEREZ |
| HOSPITAL INFANTIL DE MORELIA | MARTINA GUADALUPE BOLAÑOS MONRROY |
| HOSPITAL INFANTIL DE MORELIA | ARGELIA VEGA CAZARES |
| HOSPITAL INFANTIL DE MORELIA | BRENDA GARCIA ESTRADA |
| HOSPITAL INFANTIL DE MORELIA | CECILIA GARCÍA RUIZ DE CHAVEZ |
| HOSPITAL INFANTIL DE MORELIA | JOSÉ LUIS MARTÍNEZ TOLEDO |
| HOSPITAL INFANTIL DE MORELIA | LOURDES NIETO ARREYGUE |
| HOSPITAL INFANTIL DE MORELIA | MA.DOLORES RAUDA COVARRUBIAS |
| HOSPITAL INFANTIL DE MORELIA | MA.ELENA VARGAS ARÉVALO |
| HOSPITAL INFANTIL DE MORELIA | MARIA DE LOURDES SÁNCHEZ CRUZ |
| HOSPITAL DE ESPECIALIDADES PEDIÁTRICAS DE CHIAPAS | ENID ALEJANDRA NAVA RUÍZ |
| HOSPITAL DE ESPECIALIDADES PEDIÁTRICAS DE CHIAPAS | GRISELDA GARCIA MORALES |
| HOSPITAL DE ESPECIALIDADES PEDIÁTRICAS DE CHIAPAS | MANUEL PÉREZ GOMEZ |
| HOSPITAL CLÍNICA NOVA | SANDRA ELISA MARTÍNEZ GUTIÉRREZ |
| HOSPITAL GENERAL DE MÉRIDA YUCATÁN “DR. AGUSTÍN O´HORÁN” | GENNY MARGARITA MENDEZ GRAJALES |
| HOSPITAL GENERAL DE MÉRIDA YUCATÁN “DR. AGUSTÍN O´HORÁN” | ROSAURA PALMA PECH |
| HOSPITAL REGIONAL TIPO B, DE ALTA ESPECIALIDAD BICENTENARIO DE LA INDEPENDENCIA. | BEATRIZ GARCIA GUZMAN |
| HOSPITAL REGIONAL TIPO B, DE ALTA ESPECIALIDAD BICENTENARIO DE LA INDEPENDENCIA. | IRIS YAZMIN HERNANDEZ CÓRDOBA |
| HOSPITAL REGIONAL TIPO B, DE ALTA ESPECIALIDAD BICENTENARIO DE LA INDEPENDENCIA. | NORMA ALAVEZ RAMÍREZ |
| HOSPITAL REGIONAL TIPO B, DE ALTA ESPECIALIDAD BICENTENARIO DE LA INDEPENDENCIA. | SILVIA GUTIÉRREZ RODRÍGUEZ |
| HOSPITAL REGIONAL TIPO B, DE ALTA ESPECIALIDAD BICENTENARIO DE LA INDEPENDENCIA. | TALIA PÉREZ VICELIS |
| HOSPITAL ÁNGELES DE MORELIA | JORGE ALEJANDRO VÁZQUEZ NARVÁEZ |
| HOSPITAL ÁNGELES DE MORELIA | ALDO RAFAEL SILVA GAMIÑO |
| HOSPITAL ÁNGELES DE MORELIA | GABRIELA CHIO ORTIZ |
| HOSPITAL ANGELES MORELIA | CECILIA GUERRERO ALMEIDA |
| HOSPITAL ÁNGELES DE MORELIA | IRVIN EDUARDO JACOME GALARZA |
| HOSPITAL PARA EL NIÑO DE TOLUCA, INSTITUTO MATERNO INFANTIL DEL ESTADO DE MÉXICO | LAURA BEATRIZ MEJIA CABALLERO |
| HOSPITAL PARA EL NIÑO DE TOLUCA, INSTITUTO MATERNO INFANTIL DEL ESTADO DE MÉXICO | NATALI ROBLES ORDÓÑEZ |
| HOSPITAL REGIONAL DE ALTA ESPECIALIDAD DEL BAJÍO | JAVIER MOISÉS CASTELLANOS MARTÍNES |
| HOSPITAL REGIONAL DE ALTA ESPECIALIDAD DEL BAJÍO | MARIANA GIL VELOZ |
| HOSPITAL GENERAL LAZARO CARDENAS DEL RIO | NORBERTA VIANEY NAVARRO VARGAS |
| HOSPITAL GENERAL LAZARO CARDENAS DEL RIO | SILVIA INES RUIZ RODARTE |
| HOSPITAL GENERAL LAZARO CARDENAS DEL RIO | VICTORIA ELENA TERRAZAS LARA |
| DEPARTAMENTO DE MICROBIOLOGIA DEL SANATORIO LA LUZ | ALAN XAVIER HERNÁNDEZ FRANCO |
| DEPARTAMENTO DE MICROBIOLOGIA DEL SANATORIO LA LUZ | ANA LUISA HERNANDEZ SILVA |
| HOSPITAL DE ALTA ESPECIALIDAD DE VERACRUZ | MARISOL MANQIRUEZ REYES |
| HOSPITAL PARA EL NIÑO POBLANO | LUCIA PEREZ RICARDEZ |
| HOSPITAL PARA EL NIÑO POBLANO | MARICRUZ GUTIERREZ BRITO |
| HOSPITAL PARA EL NIÑO POBLANO | YIRLA CITLALI NAVA PACHECO |
| HOSPITAL DR. JESUS GILBERTO GOMEZ MAZA | ALEJANDRO VERA DOMINGUEZ |
| HOSPITAL DR. JESUS GILBERTO GOMEZ MAZA | ELVIRA RAMIREZ COBAXIN |
| HOSPITAL DR. JESUS GILBERTO GOMEZ MAZA | JOSUE GOMEZ ESPINOSA |
| HOSPITAL DR. JESUS GILBERTO GOMEZ MAZA | LUIS ALBERTO RUIZ GAMBOA |
| HOSPITAL DR. JESUS GILBERTO GOMEZ MAZA | SANDRA BELTRAN SILVA |
| CENTENARIO HOSPITAL MIGUEL HIDALGO | CARMEN LUCRECIA RAMOS MEDELLÍN |
| CENTENARIO HOSPITAL MIGUEL HIDALGO | CESAR ADAME ÁLVAREZ |
| CENTENARIO HOSPITAL MIGUEL HIDALGO | LUCILA MARTÍNEZ MEDINA |
| CENTENARIO HOSPITAL MIGUEL HIDALGO | MARIO GONZALEZ GAMEZ |
| CENTENARIO HOSPITAL MIGUEL HIDALGO | MORA JIMENEZ ISELA |
| CENTENARIO HOSPITAL MIGUEL HIDALGO | RICARDO GARCÍA ROMO |
| LABORTORIO DIPROMI | SANDRA MARÍA SUÁREZ MORENO |
| HOSPITAL GENERAL CON ESPECIALIDADES JUAN MARÍA DE SALVATIERRA | ANTONIA SALAS RAMIREZ |
| HOSPITAL GENERAL CON ESPECIALIDADES JUAN MARÍA DE SALVATIERRA | CONSUELO CRUZ GARCIA |
| HOSPITAL GENERAL CON ESPECIALIDADES JUAN MARÍA DE SALVATIERRA | LILIAN SOTELO PEREZ |
| HOSPITAL REGIONAL DELICIAS | VLADIMIR LINKS ESTUPIÑON |
| HOSPITAL REGIONAL DELICIAS | ANA JULIA GARCIA MEDINA |
| HOSPITAL DE ESPECIALIDADES PEDIÁTRICO DE LEÓN | ALEXANDRA ALCARAZ DELAROSA RODRÍGUEZ |
| HOSPITAL DE ESPECIALIDADES PEDIÁTRICO DE LEÓN | WANANI ESTACIHUATL LICEA GARCÍA |
| HOSPITAL ÁNGELES DE CHIHUAHUA | CLAUDIA JANETH AGUILERA ACOSTA |
| HOSPITAL ÁNGELES DE CHIHUAHUA | ELMA URBINA RODRÍGUEZ |
| GALENIA HOSPITAL | CHRISTEL YANELI COUOH CAN |
| GALENIA HOSPITAL | ENRIQUE AMBROCIO GARCÍA |
| GALENIA HOSPITAL | FRANCISCO JAVIER LÓPEZ ALVAREZ |
| GALENIA HOSPITAL | GABRIELA RODRÍGUEZ ABREGO |
| GALENIA HOSPITAL | GEORGINA HERRERA MARTÍNEZ |
| GALENIA HOSPITAL | JOSÉ EDUARDO MENDEZ CRESPO |
| HOSPITAL MATERNO INFANTIL "MORELOS CHETUMAL Q ROO. | FELIPE VAZQUEZ MORENO |
| HOSPITAL MATERNO INFANTIL "MORELOS CHETUMAL Q ROO. | GLORIA EMINA DZUL CORTES |
| HOSPITAL MATERNO INFANTIL "MORELOS CHETUMAL Q ROO. | JUAN EZEQUIEL AGUILAR MUCIÑO |
| HOSPITAL MATERNO INFANTIL "MORELOS CHETUMAL Q ROO. | MONICA ISABEL MENDOZA CANO |
| HOSPITAL MATERNO INFANTIL "MORELOS CHETUMAL Q ROO. | VERÓNICA GUADALUPE TAH GONZÁLEZ |
| HOSPITAL GENERAL DE ZONA 21 TEPATITLAN DE MORELOS | CLAUDIA LIZBETH GARCÍA CARCÍA |
| CENTRO UNIVERSITARIO DE SALUD, U.A.N.L LABORATORIO PUEBLO NUEVO | DIANA ISELA PERALTA CONTRERAS |
| CENTRO UNIVERSITARIO DE SALUD, U.A.N.L LABORATORIO VICENTE GUERRERO | RUTH FLORES LOPEZ |
| CENTRO UNIVERSITARIO DE SALUD, U.A.N.L | ELSA PERALES BERNAL |
| LABORATORIOS DEL CENTRO | ARTURO GONZÁLEZ MORENO |
| LABORATORIOS DEL CENTRO | GLORIA ARACELI MENDOZA BALLESTEROS |
| LABORATORIO DE ANALISIS BIOQUÍMICO CLÍNICOS "LOUIS PASTEUR" | ANASTACIO ROMERO SANTAMARÍA |
